# The Future Prospects of Testis Cancer in the World

**Published:** 2017-12

**Authors:** Mahin GHAFARI, Mahdi MOHAMMADIAN, Ali Asghar VALIPOUR, Abdollah MOHAMMADIAN-HAFSHEJANI

**Affiliations:** 1. Dept. of Public Health, School of Health, Shahrekord University of Medical Sciences, Shahrekord, Iran; 2. Dept. of Health and Social Medicine, School of Medicine, Dezful University of Medical Sciences, Dezful, Iran; 3. Dept. of Public Health, School of Public Health, Abadan School of Medical Sciences, Abadan, Iran; 4. Dept. of Epidemiology and Biostatistics, Isfahan University of Medical Sciences, Isfahan, Iran; 5. Dept. of Epidemiology and Biostatistics, School of Public Health, Tehran University of Medical Sciences, Tehran, Iran

## Dear Editor-in-Chief

Testicular cancer (TC) remains as a relatively infrequent tumor type, considered about 1% of all men cancers worldwide (1). In the recent years, the incidence of TC has a growing trend, so in many western societies, the incidence of TC is about doubling detected since the 1960s. In 2012, maximum and minimum of Age-Standardized Incidence Rates (ASIR) of TC in the world, observed in western Europe (ASIR=8.7 per 100000) and in Africa (ASIR= 0.4 per 100000), respectively (2). TC may seem at every age group but tend to happen in three separate age groups: 0 to 10 yr (infants and children), 15 to 40 yr (young adults) and over 60 yr (older adults) (3, 4). This tumor develops rapidly, so in 20 to 30 d tumor size is doubled and has a great risk of metastatic extent (5). Nonetheless, when it is detected and treated in early stages of disease, TC is treatable, so in developed countries, five years survival rate of disease is over 95% (6). However, survival rate of TC in developing countries is low, therefore, in the study that conducted in Tanzania, five years survival rate of TC was 22.2% (7).

In GLOBOCAN project, the estimated number of new cases and deaths of TC in a region or country in 2015, 2020, 2025, 2030 and 2035 is calculated by multiplying the age-specific rates estimated for 2012, by the corresponding expected population for 2015, 2020, 2025, 2030 and 2035. Based on GLOBOCAN project in the world in 2012, the number of 55266 new cases of TC was observed that among them 52305 cases (94.64%) were in the age group below 65 yr and 2961 cases (05.35%) were in the age group 65 yr and older. Moreover, in this year, the number of 10351 cases of death were observed that among them 7790 cases (75.25%) were in the age group below 65 yr and 2561 cases (24.75%) were in the age group 65 yr and older. According to the predictions in the years 2015, 2020, 2025, 2030 and 2035, the number of new cases of TC in the world respectively is equal to 57209, 60007, 62903, 65827 and 68351 cases. Besides, the number of deaths of TC respectively be equal to10904, 11790, 12781, 13847 and 14886 (Table 1).

**Table 1:** Predict the number of incidence and mortality of testis cancer in the world from 2012–2035

***Year***	***Predict number of new cases and deaths of TC***						***Demographic change***					
	**Ages < 65 (yr)**		**Ages >= 65 (yr)**		**Total**		**Ages < 65 (yr)**		**Ages >= 65 (yr)**		**Total**	
	**New cases**	**Deaths**	**New cases**	**Deaths**	**New cases**	**Deaths**	**New cases**	**Deaths**	**New cases**	**Deaths**	**New cases**	**Deaths**
2012	52305	7790	2961	2561	55266	10351	R	R	R	R	R	R
2015	53958	8097	3251	2807	57209	10904	1653	307	290	246	1943	553
2020	56169	8491	3838	3299	60007	11790	3864	701	877	738	4741	1439
2025	58384	8887	4519	3894	62903	12781	6079	1097	1558	1333	7637	2430
2030	60490	9233	5337	4614	65827	13847	8185	1443	2376	2053	10561	3496
2035	62160	9522	6191	5364	68351	14886	9855	1732	3230	2803	13085	4535

Population forecasts were extracted from the United Nations, World Population Prospects, the 2012 revision. Numbers are computed using age-specific rates and corresponding populations for 10 age-groups. GLOBOCAN 2012 (IARC) - 15.3.2016. Demographic changes were computed for years 2015–2035, based on the number of TC in the World in 2012 as a Reference group(R)

However, the number of incidence and mortality of TC will be increasing trend, but this increase will be more in the age group 65 yr and older. Thus, diagnosis and treatment of patients with TC in the early stages of disease lead to improving the quality of life and survival rate of patients.
